# The α2CDel322-325 adrenergic receptor polymorphism is not associated with heart failure due to idiopathic dilated cardiomyopathy in black Africans

**Published:** 2008-02

**Authors:** J Du Preez, LO Matolweni, BM Mayosi, J Greenberg, P Mntla, AA Adeyemo

**Affiliations:** Department of Medicine, Groote Schuur Hospital and University of Cape Town, Cape Town; Medical Research Council/University of Cape Town Human Genetics Research Unit, Division of Human Genetics, Department of Clinical Laboratory Sciences, University of Cape Town, Cape Town; Department of Medicine, Groote Schuur Hospital and University of Cape Town, Cape Town; Medical Research Council/University of Cape Town Human Genetics Research Unit, Division of Human Genetics, Department of Clinical Laboratory Sciences, University of Cape Town, Cape Town; Department of Medicine, Groote Schuur Hospital and University of Cape Town, Cape Town; Medical Research Council/University of Cape Town Human Genetics Research Unit, Division of Human Genetics, Department of Clinical Laboratory Sciences, University of Cape Town, Cape Town; Department of Cardiology, Dr George Mukhari Hospital and University of Limpopo, Tshwane; National Human Genome Center, Genetic Epidemiology Unit, Department of Microbiology, Howard University, Washington, DC, USA

## Abstract

**Background:**

A four-amino acid deletion was identified within the α_2C_-adrenergic receptor (α_2C_Del322-325) that, when homozygous, increases the risk of heart failure in African-Americans nearly six-fold. We hypothesised that homozygosity for the α_2C_Del322-325 polymorphism may be a risk factor for heart failure due to idiopathic dilated cardiomyopathy (DCM) in black South Africans.

**Methods:**

The α_2C_Del322-325 polymorphism was genotyped in 37 patients with heart failure and 34 controls, all of black African ancestry. Genotyping was performed by a size-fractionation assay.

**Results:**

The patients studied ranged in age from 21 to 79 years with a mean age of 50 years, and 62% were male. No significant difference was observed in homozygosity for the α_2C_Del322-325 polymorphism or in allele and genotype frequencies between patients and controls. The frequency of the allele containing the deletion was 0.54 in cases and 0.53 in controls. The genotype frequencies in the patients were consistent with those of the controls (*p* = 0.56).

**Conclusions:**

Homozygosity for the α_2C_Del322-325 polymorphism is not associated with an increased risk for heart failure due to idiopathic DCM in black South Africans.

## Summary

Dilated cardiomyopathy (DCM) is a major cause of congestive heart failure in Africa.^1^ Thirty per cent of cases are familial, illustrating the importance of genetic factors in the aetiology of this disease.^2^ A genetic variant was identified that, when homozygous, increases the risk of heart failure in African-Americans nearly six-fold.^3^ The variant, a 12bp in frame deletion within the α_2C_-adrenergic receptor (α_2C_-AR) gene, results in the loss of amino acids 322−325 (α_2C_Del322-325). The function of α_2C_-AR is to regulate the release of norepinephrine from cardiac sympathetic nerves through negative feedback. The α_2C_Del322-325 AR variant shows decreased function, which results in increased synaptic norepinephrine release. The association between the α_2C_Del322-325 AR polymorphism and heart failure is biologically plausible, as sustained cardiac adrenergic stimulation has been implicated in the development and progression of heart failure.^3^

We hypothesised that homozygosity for this variant may be a risk factor for heart failure due to idiopathic dilated cardiomyopathy in black South Africans.

## Methods

Patients of black African ancestry, who were diagnosed with heart failure due to idiopathic DCM, were recruited from Groote Schuur Hospital in Cape Town and Dr George Mukhari Hospital in Tshwane. The diagnosis of idiopathic DCM was made after comprehensive investigation to exclude a secondary cause of heart failure; cardiac catheterisation was performed in patients with a history suggestive of coronary artery disease and/or risk factors for coronary disease.

The patients provided informed consent for participating in genetic studies of cardiomyopathy, and the studies were approved by the Research Ethics Committee of the University of Cape Town. The control group, which was matched for ethnic background, was recruited from the Eye Clinic at Groote Schuur Hospital and had no history of cardiac disease, as previously reported.^4^ The racial classification of the cases and controls was by self report.

Genotyping for the α_2C_Del322-325 polymorphism was performed in 37 patients and 34 controls. The region of interest was amplified from genomic DNA using oligonucleotides 5′-TGCGTCCCCGACTACCGAAA-3′ and 5′-FAMTGACCACAGCCAGCACAAAGG-3′. Amplicons were size-fractionated on an ABI Prism® 3100 genetic analyzer (Applied Biosystems). The genotype and allele frequencies in cases and controls were compared.

The χ^2^ test was used to evaluate the association between heart failure and genotype. This was an exploratory study; hence it was not designed to maximise power to detect an association. However, given the frequency of the deletion variant in the study, the study had 91, 84 and 69% power to detect a significant association at a 95% significance level for a genotypic relative risk of heart failure of five, four and three, respectively, under an additive model (the reported increased risk is five-fold for the association of the polymorphism with heart failure in black subjects).

## Results

The 37 patients studied ranged in age from 21 to 79 years with a mean age at diagnosis of 48 (± 16) years, and 25 of the patients (68%) were male. The mean left ventricular ejection fraction was 36% (± 9), and 29 patients (78%) underwent cardiac catheterisation to exclude coronary artery disease. All patients were on standard medication for symptomatic heart failure, and two had a past history of hypertension. The HIV status of the cases was not determined in this series.

The distribution of allele frequencies was in Hardy-Weinberg equilibrium (*p* = 0.734). There was no significant association between the α2CDel322-325 polymorphism and heart failure under an additive model (χ^2^ 5 0.018, *p* = 0.894), a dominant model (χ^2^ = 0.177, *p* = 0.674), or a recessive model (χ^2^ = 0.348, *p* = 0.556); the recessive model is equivalent to comparing deletion homozygotes to the rest.

No significant difference was observed in allele and genotype frequencies of the α_2C_Del322-325 polymorphism between cases and controls (see Table 1). The frequency of the allele containing the deletion was 0.54 in cases and 0.53 in controls compared to 0.41 in black controls in the Small study.^3^

**Table 1.** Distribution Of α_2C_Del322-325 AR Variant In Patients With Heart Failure Due To Idiopathic Dilated Cardiomyopathy (Cases) And Controls

		*Genotype frequency n/total n (%)*		*Allele frequency of deletion variant*
	*Del/Del*	*WT/Del*	*WT/WT*	**
Cases	11/37 (29.7)	18/37 (48.6)	8/37 (21.6)	0.54
Controls	8/34 (23.5)	20/34 (58.8)	6/34 (17.6)	0.53

## Discussion

In this study no association was found between the α_2C_Del322-325 polymorphism and heart failure due to idiopathic DCM in black South Africans. Similar studies of the genetic association of the α_2C_Del322-325 polymorphism with heart failure in Japanese and Italian patients also failed to confirm the finding. 5,6 Furthermore, in a study of α_2C_Del322-325 and β1Arg389 polymorphisms and traits that are precursors of systolic heart failure [ie, increased left ventricular end-diastolic volume (EDV) or decreased left ventricular ejection fraction (LVEF)], no association was found in either the African-American or the white American patients.^7^

There are several reasons that may explain the discrepancy between our findings and the original report of an association, 3 but this is not unusual in case-control studies. First, we studied black Africans with idiopathic dilated cardiomyopathy, whereas the African-Americans studied, who have a different allele frequency to African blacks, had heart failure due to a number of causes including ischaemic heart disease. Second, it should be noted that this study was exploratory and the power to detect smaller relative risks (for many complex diseases, relative risks of 1.3 to 1.8 are typical) was quite limited. A larger study would demonstrate if this is indeed so. Finally, we used different genotyping methods that may limit the generalisability of our findings.

Despite these limitations, the results of our study suggest that the α_2C_Del322-325 polymorphism is unlikely to be a major risk factor for heart failure due to idiopathic DCM in our African patients.
